# Mycobacteriophages: An Integrated Platform for Rapid Diagnosis and Targeted Therapy of Tuberculosis Infection

**DOI:** 10.3390/microorganisms14102154

**Published:** 2026-09-24

**Authors:** Vadim V. Avdeev, Mikhail A. Vladimirsky, Platon I. Eliseev, Anastasia G. Samoilova, Natalia A. Nikitina, Tatiana E. Tiulkova, Irina A. Vasilyeva

**Affiliations:** Federal State Budgetary Institution «National Medical Research Center of Phthisiopulmonology and Infectious Diseases» of the Ministry of Health of the Russian Federation (NMRC PhPI), Moscow 127473, Russia; vladimirskijma@nmrc.ru (M.A.V.); pediatrics@yandex.ru (P.I.E.); a.samoilova.nmrc@mail.ru (A.G.S.); nikitinana@nmrc.ru (N.A.N.); tulkova2006@rambler.ru (T.E.T.); nmrc@nmrc.ru (I.A.V.)

**Keywords:** tuberculosis, *Mycobacterium tuberculosis*, drug resistance, mycobacteriophage D29, phage diagnostics, phage therapy, liposomes, real-time PCR, lyophilisation, aerosol therapy, pharmacokinetics

## Abstract

The global spread of multidrug-resistant (MDR) and extensively drug-resistant (XDR) tuberculosis (TB) demands fundamentally new approaches combining diagnostic speed and therapeutic efficacy. Within the strategy of personalised medicine, the lytic mycobacteriophage D29 represents a promising tool for both phenotypic drug susceptibility testing and the targeted delivery of therapeutic agents to the infection site. We developed a two-component platform based on D29: (1) a diagnostic test system combining a rapid anti-mycobacterial susceptibility testing (AMST) module, which detects phage D29 replication in drug-resistant isolates via multiplex real-time PCR; and (2) a liposomal therapeutic formulation. Therapeutic efficacy was evaluated in C57BL/6 mice (n = 55) with experimental pulmonary tuberculosis receiving liposomal D29 either aerosolised (inhalation chamber) or intranasally. Bacterial burden (CFU/lung) was assessed at days 3 and 14 post-therapy. Pharmacokinetics were studied in 90 mice using the double-layer agar method. The diagnostic test showed an average sensitivity and specificity of 90.0% and 96.3% across nine drugs, with results available within five days. The lyophilised components and phage remained stable for 12 months. Aerosol therapy demonstrated a trend towards a higher efficacy compared to intranasal administration: the median CFU/lung at day 3 was 107 (i.e., one hundred and seven) vs. 23,500 (control 65,000); 60% of aerosol-treated animals achieved ≤200 CFU/lung (not statistically significant, *p* = 0.089). Both routes significantly outperformed controls (*p* < 0.01 and *p* < 0.05). Pharmacokinetics revealed exclusive lung tropism with peak concentrations at 5 min (1.9 × 10^3^–5.1 × 10^4^ PFU/organ) and phage levels falling below the limit of detection (<5 × 10^3^ PFU/organ) within 24 h. Infected lungs showed significantly higher phage titres than uninfected ones, indicating in vivo replication. Safety studies showed no significant toxicity, immunogenicity, or local irritation. The integrated D29-based platform represents a comprehensive, integrated diagnostic–therapeutic solution covering the full “diagnosis–therapy” cycle. The test system provides rapid phenotypic drug susceptibility profiling, while the liposomal phage demonstrates potent therapeutic activity and favourable pharmacokinetics, supporting its clinical translation.

## 1. Introduction

In terms of combined morbidity and mortality, tuberculosis (TB) has no equal among infectious diseases—its annual incidence and mortality rates remain the highest in the world. According to the World Health Organisation, in 2024, TB was the world’s leading cause of death from a single infectious agent (1.23 million), [1] surpassing viral hepatitis [2], COVID-19 [3], and HIV [4]. It should be noted that, in 2021, the Russian Federation left the list of countries with a high TB burden. In 2024, historic lows were recorded for the incidence and mortality of this deadly infection [5]. However, the situation is aggravated by the steady increase in *Mycobacterium tuberculosis* strains with rifampicin resistance (RR), multidrug resistance (MDR), extensive drug resistance (XDR), and total drug resistance: in 2024, MDR/RR-TB caused about 150,000 deaths globally, and the number of new MDR-TB cases approaches 400,000 annually [6,7]. The proportion of MDR among newly diagnosed patients in Russia rose from 13.0% in 2009 to 32.0% in 2024 [8]. Addressing this problem requires rapid diagnostic methods and drugs with novel mechanisms of action. To address this, we developed a rapid anti-mycobacterial susceptibility testing (AMST) system. The core methodological principle of this AMST system is that, if an anti-TB drug fails to kill the bacteria during early incubation, the surviving resistant cells are subsequently infected by the phage; although these bacteria remain phenotypically invisible during the initial days of culture, their presence is revealed indirectly by assaying the replicated bacteriophage DNA via real-time PCR.

Current drug susceptibility testing relies on two main approaches: culture-based and molecular-genetic methods. Culture-based methods (the gold standard) do not allow rapid results due to the structure of *M. tuberculosis* and the peculiarities of their growth on nutrient media. The waiting time for results extends to 28–42 days, delaying the prescription of adequate personalised chemotherapy based on *M. tuberculosis* drug susceptibility test results [9]. Automated systems such as Bactec MGIT 960 shorten the drug susceptibility testing time to 14 days. However, even this period is insufficient for the timely initiation or adjustment of chemotherapy [10]. The delayed prescription of anti-TB drugs (ATDs) promotes the selection and emergence of new mutations associated with drug resistance (DR), as well as the accumulation of DR strains. Nucleic acid amplification tests (NAATs) provide results within hours but are limited to detecting known mutations and fail to identify resistance due to novel or rare genetic mutations, which are typically detected by phenotypic methods, leading to potential discrepancies between genotypic and phenotypic drug susceptibility results [11,12,13]. To obtain comprehensive information on mutations, next-generation sequencing (NGS) is used; however, the results typically require several days when clinical samples are analysed and several weeks when cultured isolates are used. Rapid diagnostic approaches, including isothermal amplification (loop-mediated isothermal amplification—LAMP [14] and recombinase polymerase amplification—RPA [15]) and DNA visualisation methods, are under development and lack widespread clinical implementation [16]. Prokaryotic adaptive immune systems called CRISPR-Cas systems have transformed genome editing by allowing for precise genetic alterations through targeted DNA cleavage. This new direction in genetic research on the natural bacterial defence system of “clustered regularly interspaced short palindromic repeats” enables the rapid identification of gene mutations [17]. Some CRISPR-associated proteins (Cas) possess trans-cleavage activity, opening new possibilities for applying CRISPR/Cas in the diagnosis of TB drug resistance [18]. Despite the speed of information acquisition, molecular methods are limited to known mutations and do not account for the complex resistance mechanisms of living systems. Thus, there is a need for diagnostic systems that combine the speed of molecular methods with the phenotypic reliability of culture-based techniques. The ideal diagnostic tool should indicate not only the presence of resistance genes but also the actual viability of bacteria in the presence of antibiotics. This requirement is fully met by developing test systems using mycobacteriophages, which can serve as biological reporters: if bacteria survive antibiotic exposure (indicating resistance), the phage infects and replicates within them, and this replication is rapidly detected via molecular methods such as real-time PCR.

Mycobacteriophages are viruses that selectively infect mycobacteria, a genus of slow-growing bacteria with a waxy, lipid-rich cell wall that confers resistance to many conventional antibiotics. This includes *M. tuberculosis*, the causative agent of tuberculosis. The selective affinity of mycobacteriophages for their host underpins phenotypic drug resistance (DR) testing, which reveals the actual bacterial response to a drug—unlike molecular-genetic tests that detect only known resistance mutations. The most promising direction is the use of lytic bacteriophages engineered as reporter phages: these not only lyse live *M. tuberculosis* cells but also serve as rapid indicators of drug susceptibility. Specifically, they carry a reporter gene (e.g., luciferase) that begins to glow only when the phage actively replicates inside live mycobacteria. If the bacteria are susceptible to the drug, they die, the phage does not replicate, and no signal is detected. If the bacteria are resistant, they survive, the phage replicates, and a bright signal is recorded. The genetically engineered phages TM4::GeNL, developed by an international group of scientists, can determine the resistance to five drugs, including new ones (bedaquiline, and pretomanid), in just 48 h [19]. Another method, Phage-LAMP, combines phage-mediated bacterial lysis with highly sensitive DNA amplification (LAMP), allowing for the rapid and accurate detection of mycobacteria directly in clinical samples [20]. In a test with functionalised nanocrystals, phages are used to create biosensors capable of detecting *M. tuberculosis* with a high sensitivity (up to 1000 bacteria per sample) in various biological fluids (sputum and blood) in just 35 min [21]. The use of mycobacteriophage in a test system allows results to be obtained within hours, with accuracy comparable to the culture method.

The genome of the lytic phage D29 (49,136 bp) contains three genes encoding lysin A, lysin B, and holin, which hydrolyse peptidoglycan and disrupt the mycolic acid layer, leading to bacterial lysis [22,23,24,25,26]. This property of the lytic mycobacteriophage D29, which is active against *M. tuberculosis*, to rapidly detect viable cells and simultaneously lyse even drug-resistant isolates is used not only in diagnostic test systems but also in TB therapy. In preclinical studies, D29 showed the highest lytic activity among tested phages against clinical *M. tuberculosis* isolates [27,28]. It is important to note that the efficacy of this phage-based platform inherently depends on the clinical isolate being susceptible to mycobacteriophage D29. Therefore, our methodology incorporates a built-in quality control criterion that serves as a parallel screening for the phage replication capacity. If the phage fails to replicate in the growth control, the test is deemed invalid. This ensures that diagnostic results are applicable exclusively to isolates supporting phage replication, which also serves as a predictive biomarker for therapeutic efficacy, as in vitro phage replication is a prerequisite for successful phage therapy in vivo. Recent reviews highlight the high potential of mycobacteriophages for the diagnosis and therapy of mycobacterial infections, including those caused by non-tuberculous mycobacteria [29,30,31,32].

Phage therapy is a treatment method for bacterial infections using bacteriophages (or, simply, phages)—viruses that selectively infect and destroy bacterial cells. In the era of growing antibiotic resistance, this approach is attracting increasing attention as a promising alternative or supplement to traditional antibiotics. Phages act in a targeted manner, destroying only the pathogenic bacterium and not affecting the normal microflora of the body [33]. This does not disrupt the local microbiome (e.g., of the respiratory tract) and does not provoke the development of associated complications [34]. Phages are capable of infecting and destroying strains with total drug resistance, against which traditional antibiotics are powerless [35]. Their mechanism of action differs from that of antibiotics, so cross-resistance is absent [36]. Unlike antibiotics, phages replicate only in the presence of the target bacterium: when all target bacteria are destroyed, phages are naturally eliminated from the body because they have no host on which to replicate. Phages have no toxic effect on human or animal cells. They can be used in patients of all age groups, including newborns and pregnant women [37].

The main challenge remains the efficient delivery of the phage to the infection site—pulmonary granulomas, where the pathogen often resides in a metabolically altered state [38,39]. The encapsulation of phages in liposomes addresses this issue by protecting virions from immune neutralisation, prolonging their action, and facilitating the uptake by infected macrophages [40,41,42]. Liposomal delivery systems are being actively studied for TB therapy; they provide passive targeting to macrophages, improve intracellular delivery, and can be administered both intravenously and by inhalation [40,42]. Previous studies showed that the transport of liposome-encapsulated mycobacteriophage into macrophages is 6–8 times higher than that of free phage [43]. The first reports of successful phage application in patients with mycobacterial infections—including 20 cases of refractory non-tuberculous mycobacteriosis—confirmed the safety and potential efficacy of this approach [37]. However, no clinical trials of phage therapy for TB have been conducted to date, and significant barriers remain, including the standardisation of phage preparations and dosing regimens [44,45].

Thus, the use of lytic bacteriophages to specifically kill pathogens is considered one of the most promising avenues [46,47]. The lytic mycobacteriophage D29, which can infect and lyse both fast-growing non-tuberculous mycobacteria and *M. tuberculosis*, including drug-resistant isolates, is an ideal candidate for both diagnostic and therapeutic applications [23,27]. All the above opens the way to creating a unified platform combining diagnosis and treatment.

The accumulated evidence suggests that the traditional paradigm of “diagnosis–therapy selection–treatment” for MDR-TB operates with significant delays and losses. A paradigm shift is urgently needed: the diagnostic tool should not only identify the pathogen but also predict the effectiveness of specific drugs and even perform a therapeutic function. This is precisely the potential carried by bacteriophages. Unlike antibiotics, they evolve alongside bacteria, possess a narrow specificity, and can be engineered as binary agents: a reporter for the rapid susceptibility analysis and a lytic effector for pathogen destruction. Phage D29, which actively lyses *M. tuberculosis* in vitro, has already shown itself to be a promising candidate for implementing this dual function. The development of a platform based on it could radically change the clinical management pathway for patients with resistant TB.

While the individual components of this approach—mycobacteriophage D29, liposomal encapsulation, and phage-based drug susceptibility testing (DST)—have been previously described in the literature, the novelty of the present study lies in their strategic integration into a unified, closed-loop ‘diagnosis-to-therapy’ platform. Rather than developing entirely new biological constructs, this work demonstrates the feasibility of using a single, well-characterised agent (phage D29) to, first, rapidly determine the phenotypic drug susceptibility profile of clinical *M. tuberculosis* isolates, and subsequently deploy the same agent as a targeted, liposome-encapsulated therapeutic. This integrated approach aims to bridge the critical gap between rapid diagnostics and personalised, targeted treatment for drug-resistant tuberculosis.

Given the above, the aim of this comprehensive work was to develop and comprehensively evaluate an integrated platform based on mycobacteriophage D29, comprising the following: (1) a diagnostic module—a test system for the accelerated phenotypic determination of the drug susceptibility of *M. tuberculosis* to a broad range of drugs; and (2) a therapeutic module—a liposomal formulation of phage D29 with the optimisation of administration routes and pharmacokinetic characterisation in an experimental TB model.

## 2. Materials and Methods

### 2.1. Diagnostic Module: The AMST System

#### 2.1.1. Biological Objects and Reagents

The lytic mycobacteriophage D29 was obtained from the collection of the National Medical Research Center of Phthisiopulmonology and Infectious Diseases. The lytic nature of the D29 strain used in this study was confirmed by whole-genome sequencing (NGS) using Illumina MiSeq (Illumina, San Diego, CA, USA), yielding an average coverage of 1089.7× (minimum coverage: 300×). Bioinformatics analysis demonstrated 100% sequence identity to the reference mycobacteriophage D29 genome (GenBank accession NC_001900.2), originally characterised by Ford et al. [26]. The reference genome lacks a complete repressor gene and integrase, confirming that D29 is a strictly lytic phage incapable of establishing lysogeny [48]. *Mycobacterium smegmatis* mc^2^155 (ATCC 700084) was used for phage propagation and titration. Clinical validation was performed on 45 clinical *M. tuberculosis* isolates provided by the Laboratory of Microbiology of the National Medical Research Center of Phthisiopulmonology and Infectious Diseases. All isolates were preliminarily identified as the *M. tuberculosis* complex and originated from various biological materials: sputum (n = 32), bronchoalveolar lavage (BAL, n = 8), lymph node tissue (n = 1), lung tissue (n = 1), joint tissue (n = 1), and pleural fluid (n = 2). *M. tuberculosis* H37Rv (ATCC 27294) was used as the reference strain for optimisation experiments. Middlebrook 7H9 liquid medium (Himedia, Mumbai, Maharashtra, India) supplemented with 10% OADC and 1 mM CaCl_2_, as well as Middlebrook 7H10 agar medium, were used for mycobacterial cultivation. Critical concentrations of first- and second-line anti-TB drugs were as follows: isoniazid (0.1 µg/mL), rifampicin (0.5 µg/mL), ethambutol (5 µg/mL), levofloxacin (1 µg/mL), moxifloxacin (0.25 µg/mL), bedaquiline (1 µg/mL), linezolid (1 µg/mL), delamanid (0.06 µg/mL), and clofazimine (1 µg/mL). The AMST protocol aligns with the WHO operational handbook on tuberculosis (Module 3: diagnosis, 2025, Table 2.9) [49], with minor adaptations for the 7H9 liquid medium format (e.g., utilizing 0.5 μg/mL rifampicin), which has been locally validated for this specific assay setup. Lyophilised anti-TB drugs were utilised to ensure long-term reagent stability and eliminate cold-chain requirements, facilitating decentralised testing.

#### 2.1.2. Preparation of Bacterial Suspension

*M. tuberculosis* colonies from Löwenstein–Jensen medium were collected with a sterile loop, avoiding agar carryover. The bacterial mass was transferred to a tube with physiological saline and glass beads (3 mm), vortexed for 1 min, and allowed to settle for 30 min. The upper homogeneous part was taken and adjusted to McFarland standard 1.0 using a densitometer, corresponding to ~3 × 10^8^ CFU/mL.

#### 2.1.3. Testing Protocol

Into wells of 24-well culture plates containing lyophilised anti-TB drugs at critical concentrations in final volume, 800 µL of liquid medium “MB7H9+” (Middlebrook 7H9 + 10% OADC + 1 mM CaCl_2_) was added. Then, 200 µL of the prepared *M. tuberculosis* suspension was added to each well. For each isolate, a growth control well (GC) without drugs was included. Plates were incubated at 37 °C for 48 h. Then, 50 µL of phage D29 (10^8^ PFU/mL) was added to all wells, including GC, and incubation continued for another 48 h. A phage control (PC) sample (original phage without culture) was prepared in parallel.

#### 2.1.4. DNA Extraction and Multiplex Real-Time PCR

On day 5, the contents of the wells were transferred to microcentrifuge tubes, centrifuged (13,000 rpm, 10 min), the supernatant discarded, and the pellet resuspended in 200 µL of deionised water and subjected to thermal lysis (95 °C, 30 min). After centrifugation, the supernatant was used as PCR template. Reactions (25 µL) were run on a CFX96 amplifier (Bio-Rad Laboratories, Hercules, CA, USA) with 10× PCR buffer (500 mM KCl, Tris-HCl pH 8.8), 25 mM MgCl_2_, dNTP mixture (250 µM each), hot-start Taq polymerase (2.5 U), specific primers and probe for D29 detection (D29-F: agccgatcagaagcacgggc; D29-R: agcggctcttaggaggggcc; probe Pb_D29_up_FAM: FAM-agccacgaactcgcgacccacgg-RTQ1), and a commercial primer/probe mixture for the *M. tuberculosis regX3* gene (ROX channel) (Syntol, Moscow, Russia). Amplification protocol: 95 °C for 2 min; and 40 cycles of 95 °C for 15 s and 63 °C for 40 s (fluorescence data collection). The D29-F and D29-R primer sequences and the Pb_D29_up_FAM probe were designed according to Pholwat et al. [50] based on the reference genome NC_001900.2 [26].

#### 2.1.5. Interpretation and Quality Control

The following quality control checks were implemented to ensure the reliability of the PCR amplification and the validity of the ΔCt calculations.

Results were considered valid if two conditions were met:

(i) Internal consistency check: Ct (GC) ≤ Ct (any drug) + 3. This criterion verifies that the Growth Control (GC) is not an anomalous outlier and that phage replication in the GC is consistent with replication in drug-containing wells where bacteria are expected to survive (resistant isolates). If this condition is not met, the entire test is deemed invalid due to potential technical errors.
(ii) Phage viability and replication capacity: ΔCt (phage) = Ct (PC) − Ct (GC) > 3 (corresponding to at least a 10-fold increase in phage DNA). This is the primary and most stringent criterion, which simultaneously confirms both of the following: (a) the viability of the phage preparation, and (b) the ability of the specific tested isolate to support phage replication. If this condition is not met, the isolate does not support phage replication, and diagnostic results for that isolate are not applicable.

For each drug well, ΔCt (drug) = Ct (drug) − Ct (GC) was calculated. Susceptible (S) was defined as ΔCt ≥ 3, resistant (R) as ΔCt < 3.

H37Rv *M. tuberculosis* strain was used as control.

The built-in QC criterion (condition ii) ensures that diagnostic results are applicable exclusively to isolates supporting phage D29 replication. This screening serves a dual purpose, confirming diagnostic eligibility and serving as a predictive biomarker for therapeutic efficacy, as in vitro phage replication is a prerequisite for successful phage therapy in vivo.

Worked example: If Ct (PC) = 38.0, Ct (GC) = 34.0, and Ct (Isoniazid well) = 39.0:

(1) Internal consistency (condition i): Ct (GC) = 34.0, Ct (Isoniazid) + 3 = 39.0 + 3 = 42.0. Since 34.0 ≤ 42.0, the GC is consistent and the assay is valid.
(2) Phage viability and replication capacity (condition ii): ΔCt (phage) = Ct (PC) − Ct (GC) = 38.0 − 34.0 = 4.0. Since 4.0 > 3, the phage is viable and successfully replicated in the tested isolate.
(3) Interpretation: For Isoniazid, ΔCt = Ct (drug) − Ct (GC) = 39.0 − 34.0 = 5.0. Since 5.0 ≥ 3, the isolate is classified as Susceptible to Isoniazid.

#### 2.1.6. Lyophilisation and Stability Assessment

Anti-TB drugs were lyophilised directly in 24-well plates using a stabiliser based on Ficoll and mannitol. Concentrations of 1%, 5%, 15%, and 50% were screened; 15% was optimal, providing good fixation and no detachment upon storage. Lyophilisation programme: −30 °C for 60 min, −80 °C for 30 min, −30 °C for 600 min, and +25 °C for 180 min. Activity of lyophilised drugs was confirmed on H37Rv in comparison with liquid forms.

For phage lyophilisation, trehalose at 50%, 20%, and 10% (with additional molar variants 1M, 0.8M, and 0.5M) was tested; 20% was optimal. Stability of the liquid phage preparation was monitored at +2…+8 °C for 48 weeks. Activity was determined every 4 weeks by double-layer agar with *M. smegmatis*.

#### 2.1.7. Comparative Testing with the Reference Method

A prospective blinded study was conducted: the same 45 clinical isolates were tested in parallel with the developed test system and the Bactec MGIT 960 automated system (Becton, Dickinson and Company, Sparks, MD, USA) according to the manufacturer’s instructions. The results obtained with the AMST system were evaluated against the reference method (Bactec MGIT 960) as follows: true resistant (TP)—the isolate was classified as resistant by both the test system and the reference method; true susceptible (TN)—the isolate was classified as susceptible by both methods; false resistant (FP)—the test system classified the isolate as resistant, while the reference method classified it as susceptible (false-positive result); and false susceptible (FN)—the test system classified the isolate as susceptible, while the reference method classified it as resistant (false-negative result). For each drug, diagnostic sensitivity (TP/(TP + FN) × 100%) and specificity (TN/(TN + FP) × 100%) were calculated.

### 2.2. Therapeutic Module: Liposomal Mycobacteriophage D29

#### 2.2.1. Production and Purification of Mycobacteriophage D29

For phage propagation, *M. smegmatis* was grown in Middlebrook 7H9 with 1 mM CaCl_2_ to OD = 1.0. Yeast extract (5%, 1 mL per 100 mL) was added, and the culture was incubated in a shaker (70 rpm, 37 °C) for 24 h. Then, 5 mL of phage (10^5^–10^8^ PFU/mL) was added and incubation continued for another 24 h. The suspension was centrifuged (3000 rpm, 15 min), and the supernatant filtered through 0.22 µm. The titre was determined by double-layer agar.

Purification from bacterial lysates was performed by ion-exchange chromatography on Macro-Prep DEAE Media (BioRad, USA) using a BioLogic LP chromatograph. Phage preparation (50–70 mL) was loaded onto the column equilibrated with phage buffer (0.01 M Tris-HCl, 0.1 M MgSO_4_, 0.1 M NaCl, and pH 7.5). After elution of the first peak, NaCl concentration was raised to 1 M, and the second peak containing phage was collected. The purified phage was dialysed against 0.1 M phage buffer (24 h, 4 °C) and sterilised by filtration (0.22 µm). Activity of purified phage was 10^8^–10^9^ PFU/mL.

#### 2.2.2. Preparation of Liposomal Phage by Extrusion

A lipid film was prepared from 30 mg soy phosphatidylcholine (Sigma-Aldrich, St. Louis, MO, USA) and 10 mg cholesterol (Sigma-Aldrich) dissolved in 3 mL of 95% ethanol and dried on a rotary evaporator. The film was hydrated with 3 mL of purified phage and vortexed for 5 min. Multilamellar liposomes were sequentially extruded (20 passes) through polycarbonate filters: a single pass through a 5 µm filter, followed by 19 passes through a 0.45 µm filter, and a final single pass through a new 0.45 µm filter (Merck Millipore, Burlington, MA, USA). The sterile preparation was stored at 4 °C. Liposome size and polydispersity index (PDI) were controlled by dynamic light scattering (DLS) using a Malvern Zetasizer (Malvern Panalytical Ltd., Malvern, Worcestershire, UK), as detailed in our recent work [51]. Phage encapsulation efficiency was determined by the difference in phage DNA concentrations in the supernatant and pellet after centrifugation (14,000 rpm, 30 min) using real-time PCR, yielding an initial encapsulation efficiency of 34.1 ± 1.5% [51].

#### 2.2.3. Experimental Animals and Infection Model

A total of 45 mice were used for the therapeutic efficacy experiments (15 per group), and 90 mice were used for the pharmacokinetic study. The sample size was chosen based on standard practice in similar preclinical studies; no formal a priori power calculation was performed, but the numbers are consistent with those typically used in murine TB models. Experiments were performed on inbred C57BL/6 mice (body weight 18–22 g). Animals were kept under standard vivarium conditions (12/12 h light cycle, free access to water and feed). All animal procedures were performed at the Scientific Center for Applied Microbiology (Obolensk, Moscow Region) and were approved by its Ethics Committee (protocol N 634-n\22, 15 August 2022).

TB infection was induced by intranasal instillation of 20 µL of *M. tuberculosis* H37Rv suspension (1.5 × 10^5^ CFU/mouse) under light anaesthesia (xylazine/ketamine (Rometar 2%, Spofa, Prague, Czech Republic), 200 µL/mouse). On day 30, one animal was sacrificed to confirm infection. Aerosol infection was performed using a Glas-Col^®^ 099C A4224 device (Glas-Col, LLC, Terre Haute, IN, USA) (target dose ~100 CFU/mouse).

#### 2.2.4. Validation of Aerosol Delivery System

Prior to vivo experiments, the aerosol delivery technique was validated to ensure phage viability and liposome integrity post-nebulisation. A compression nebuliser (OMRON NE-C300 Complete, OMRON Corporation, Kyoto, Japan; setting 3, 0.3 mL/min, particle size 3 µm) was used for all validation experiments.

Phage viability assessment: Purified phage D29 (12 mL in phage buffer: 0.01 M Tris-HCl, 0.1 M MgSO_4_, 0.1 M NaCl, and pH 7.5) was passed through the nebuliser and the nebulised fraction was collected. The phage titre was determined by the standard double-layer agar plaque assay using *M. smegmatis* mc^2^155 as the host strain. Three independent experiments were performed to assess phage titre loss.

Liposome integrity assessment: Liposomal phage D29 (0.45 µm, prepared by extrusion as described in Section 2.2.2) was similarly passed through the nebuliser under identical conditions. The integrity of liposomes post-nebulisation was evaluated by quantifying the phage encapsulation efficiency using quantitative real-time PCR (qPCR) with D29-specific primers (D29-F, and D29-R) and probe (Pb_D29_up_FAM), as described in Section 2.1.4. The encapsulation efficiency was calculated as the percentage of total phage DNA that remained associated with intact liposomes.

#### 2.2.5. Titration of Liposomal Phage Preparations

To determine the total phage titre (encapsulated and free) of the liposomal preparation or organ homogenates, the liposomes were chemically disrupted prior to the double-layer agar plaque assay to prevent interference from intact lipid vesicles, as described in our previous work [51].

Disruption protocol: The liposomal suspension sample was pre-treated with 1% Triton X-100 solution (Wuhan Servicebio Technology Co., Ltd., Wuhan, Hubei, China) for 10 min at room temperature to completely disrupt the lipid membranes and release the encapsulated phage particles.

Plaque assay: Following disruption, serial 10-fold dilutions were prepared in SM-buffer, and the titre was determined using the standard double-layer agar plaque assay with *M. smegmatis* mc^2^155 as the host strain on Middlebrook 7H10 agar. After adsorption and incubation at 37 °C for 24 h, plaques were counted.

This detergent-based method ensures complete liposome disruption without compromising phage viability or interfering with plaque formation.

#### 2.2.6. Dosing Regimens

Treatment was initiated on day 33 post-infection. Animals were randomised into three main groups (n = 15 each):

Group 1 (aerosol therapy): inhalations in the Glas-Col chamber, phage concentration 10^8^ PFU/mL, spray volume 7 mL, duration 30 min, schedule: 5 consecutive days → 2 days break → 5 consecutive days (total 10 sessions).

Group 2 (intranasal therapy): instillation of 50 µL of the preparation (10^8^ PFU/mL) into the nostrils on inspiration, twice daily with a 6-hour interval, schedule: 5 consecutive days → 2 days break → 5 consecutive days (total 20 administrations).

Group 3 (infection control): infected, untreated.

An additional group 4 (aerosol infection, n = 10) was formed for model reproducibility control.

#### 2.2.7. Efficacy Assessment

Euthanasia was performed by ether overdose at two time points:

Early effect (3 days after the last administration): 10 animals from groups 1–3 and all 10 from group 4 were sacrificed.

Delayed effect (14 days after the end of therapy): the remaining 5 animals from groups 1–3 were sacrificed.

Lungs were removed aseptically, weighed, and examined macroscopically for pathology (foci, hyperaemia, and haemorrhages). Lungs were homogenised in 2 mL of sterile physiological saline, serial 10-fold dilutions were prepared, and 100 µL of each dilution was plated onto Middlebrook 7H10 with OADC. Plates were incubated at 37 °C for 21 days, colonies counted, and results expressed as CFU/lung.

For histological analysis, fragments of nasal turbinates, trachea, and lungs were fixed in 10% neutral buffered formalin, processed on a Milestone LOGOS apparatus (Milestone S.r.l., Sorisole (BG), Italy), embedded in paraffin (Leica HistoCore Arcadia, Leica Biosystems Nussloch GmbH, Nussloch, Germany), sectioned at 3 µm on a Leica RM2245 microtome (Leica Biosystems Nussloch GmbH, Nussloch, German), stained with haematoxylin-eosin, and examined under a Leica DM3000 microscope (Leica Microsystems Wetzlar GmbH, Wetzlar, Germany).

Histological changes were evaluated using a semi-quantitative scoring system (scale 0–3): 0 = normal architecture; 1 = minimal changes (slight thickening of alveolar walls, focal non-specific changes); 2 = moderate changes (focal lymphoplasmacytic infiltration, single necrosis foci); and 3 = severe changes (diffuse infiltration, extensive necrosis). The evaluation was performed in a blinded manner by a certified pathologist unaware of group assignments.

#### 2.2.8. Pharmacokinetic Study

The study was performed on 90 mice (females, 20–25 g) divided into 7 groups (15 each). Regimens included intact and infected animals with single or multiple (5 + 2 + 5 days) intranasal or aerosol administration. Organs (lungs, liver, kidneys, spleen, mesenteric lymph nodes, and blood) were collected at 5 min, 1 h, and 24 h after the last administration (5 animals per time point). Organs were homogenised in SM buffer (100 mM NaCl, 10 mM MgSO_4_, 10 mM Tris-HCl, and pH 7.5) with addition of chloroform (100 µL) to inactivate microflora. Phage concentration was determined by the double-layer agar method with *M. smegmatis*.

#### 2.2.9. Toxicological Studies

While in vitro cytotoxicity assays on pulmonary cell lines are valuable for initial safety screening, the present study focused on in vivo toxicity assessment because it is mandatory for regulatory approval and provides a more comprehensive evaluation of systemic and local adverse effects, including immunogenicity, which cannot be fully recapitulated in cell culture.

Acute toxicity: Outbred male mice (n = 15) received a single intraperitoneal injection of the liposomal phage preparation at a volume of 5.0 mL/kg. Based on the preparation’s composition (40 mg total lipids per 3 mL) and phage titre (10^8^ PFU/mL), this administration corresponds to a lipid dose of approximately 67 mg/kg and a phage dose of 5.0 × 10^8^ PFU/kg (representing a 200-fold therapeutic dose). Controls (n = 10) received vehicle. Observation lasted 30 days with daily clinical examination, body weight, and leukocyte counts.

Chronic toxicity: Mice (n = 25) received daily intraperitoneal injections at a volume of 25 mL/kg for 20 days. This regimen corresponds to a lipid dose of approximately 330 mg/kg and a phage dose of 2.5 × 10^9^ PFU/kg. Controls (n = 10) received vehicle. Observation continued for 7 days after the last dose.

Immunogenicity: Plasma from mice after 20-day administration was incubated with phage (10^6^ PFU/mL) for 30 min at 37 °C; then, residual phage activity was determined by double-layer agar and PCR, and ELISA was performed to detect specific antibodies.

Local irritation: This was studied after a single subcutaneous injection (volume 25 mL/kg, corresponding to 330 mg/kg lipid mass and 2.5 × 10^9^ PFU/kg) with evaluation of the injection site at 3 days.

#### 2.2.10. Statistical Analysis

For diagnostic data (sensitivity/specificity), 95% confidence intervals were calculated. For therapeutic experiments, the non-parametric Mann–Whitney U test and χ^2^ test were used; differences were considered significant at *p* < 0.05. Data are presented as median (interquartile range) or mean ± standard error. Statistical analyses were performed using Statistica 10.0 (StatSoft, Inc., Tulsa, OK, USA).

## 3. Results

### 3.1. Development and Validation of the AMST System

#### 3.1.1. Prototype and Configurations

A fully functional prototype of the test system was created, consisting of five components: (1) liquid medium “MB7H9+”, (2) mycobacteriophage D29, (3) plates with lyophilised drugs, (4) PCR-RT kit, and (5) deionised water. Three configurations were developed:

- AMST-3 (INH, RIF, and EMB)—six plates, each for four samples;
- AMST-6 (Lfx, Mfx, Bq, Lzd, Dlm, and Cfz)—eight plates, each for three samples;
- AMST-9 (all nine drugs)—twelve plates, each for two samples.

This allows for a flexible testing volume depending on clinical needs.

#### 3.1.2. Component Stability

Lyophilisation with a 15% stabiliser provided the clear fixation of drugs in wells without detachment. The activity of lyophilised drugs, tested on H37Rv, was identical to the liquid forms (ΔCt ≥ 6 for all drugs, indicating susceptibility).

The monitoring of three batches of liquid phage D29 showed that the activity remained at 3–5 × 10^9^ PFU/mL for 32 weeks. From week 36, a two-to-three-fold decrease (to 1–3 × 10^9^ PFU/mL) was observed, which is not critical for the system (required working titre 10^8^ PFU/mL). A shelf life of 12 months at +2…+8 °C was established. The preliminary data on the lyophilised phage with 20% trehalose showed preserved activity for 3 months.

#### 3.1.3. Diagnostic Performance

A comparative testing of 45 clinical isolates (including various biomaterials) against the Bactec MGIT 960 reference method gave high results (Table 1). The average sensitivity across nine drugs was 90.0% (88.1% for first-line, 91.0% for second-line), and the average specificity was 96.3% (95.0% and 97.1%, respectively). For the key drugs isoniazid and rifampicin, the sensitivity exceeded 96%, which is critical for MDR detection. The sensitivity for ethambutol and bedaquiline was somewhat lower (71% and 67%), likely due to the limited number of resistant isolates in the sample (n = 31 for EMB, n = 9 for Bq) and their bacteriostatic mechanisms. The specificity reached 100% for EMB, Lfx, Mfx, and Bq, guaranteeing no false-positive results.

A representative example of the interpretation is shown in Figure 1, which presents the kinetic curves for isolate No. 32, demonstrating the resistance to several drugs and retained sensitivity to one of the tested drugs.

### 3.2. Preclinical Studies of Liposomal Mycobacteriophage D29

#### 3.2.1. Physicochemical Characterisation of Liposomal Phage

The comprehensive physicochemical characterisation of the liposomal mycobacteriophage D29 formulation used in this study was recently established and published in detail by our group [51].

For the current study, dynamic light scattering (DLS) and transmission electron microscopy confirmed a mean liposome diameter of 300–450 nm (0.3–0.45 µm) for the formulation extruded through 0.45 µm filters (Merck Millipore, USA). The polydispersity index (PDI) indicated a narrow size distribution and a homogeneous population of vesicles, which is optimal for alveolar delivery and macrophage uptake. The phage encapsulation efficiency, quantified via the qPCR-based method described previously [51], was determined to be 34.1 ± 1.5% at the initial time point (0 months).

The therapeutic dose of 10^8^ PFU/mL was defined as the titre of the original phage preparation used for liposome encapsulation. Given the encapsulation efficiency of approximately 34% [51], the actual encapsulated phage titre in the final preparation was approximately 3.4 × 10^7^ PFU/mL.

#### 3.2.2. Validation of Aerosol Delivery

The aerosol delivery system was validated to confirm that nebulisation does not significantly compromise phage viability or liposome integrity.

Phage viability: Three independent experiments demonstrated minimal phage titre loss during nebulisation. The initial phage titre ranged from 5 × 10^8^ to 7 × 10^8^ PFU/mL, and post-nebulisation titres ranged from 1 × 10^8^ to 3 × 10^8^ PFU/mL, representing a loss of approximately 0.5 log_10_ (Table 2). This minimal reduction confirms that the nebulisation process preserves the biological activity of the phage.

Liposome integrity: The encapsulation efficiency of phage D29 in liposomes decreased by only 6 percentage points following nebulisation (from 34% to approximately 28%), as determined by qPCR analysis. This indicates that the liposomal structure remains largely intact during the aerosolisation process, ensuring that the encapsulated phage is protected during delivery.

These validation data confirm that the nebulisation procedure does not significantly degrade the phage or disrupt the liposomal formulation, ensuring that the animals received the intended therapeutic dose of active, encapsulated virions.

#### 3.2.3. Efficacy of Different Administration Routes

Both intranasal (Group 3) and aerosol (Group 4) infection routes produced comparable high bacterial burdens (median CFU/lung ~65,000), confirming model reproducibility. Three days after therapy, aerosol administration (Group 1) reduced the median to 107 (0–520), while intranasal (Group 2) reduced it to 23,500 (0–151,000). The proportion of animals with low bacterial load (≤200 CFU/lung) was 60% (6/10) in Group 1 and 40% (4/10) in Group 2; none in the control. The complete absence of growth (0 CFU) was seen in 30% (3/10) of Group 1 and 20% (2/10) of Group 2. Both treatment groups were significantly different from controls (*p* < 0.01 for Group 1, *p* < 0.05 for Group 2). The direct comparison of Groups 1 and 2 showed no significant difference (U = 37, *p* = 0.089), but a trend favouring the aerosol route was observed, consistent with data from Liu et al., who demonstrated that the aerosol delivery of phage D29 provides more efficient deposition in the airways compared to intranasal administration [52].

At day 14 post-therapy, the median CFU in both treatment groups fell to 0 (IQR: Group 1 0–23, Group 2 0–57), while the control remained high (44,000, 28,000–100,000). The proportion of animals with ≤200 CFU/lung was 60% in both groups, with zero growth in 40%. These data indicate a sustained therapeutic effect and possible clearance by the immune system.

#### 3.2.4. Histological Evaluation of Local Tolerance

A histological examination of the respiratory tract tissues showed that aerosol administration preserved the normal architecture of the nasal mucosa and trachea without signs of inflammation. Intranasal administration caused the minimal deformation of glandular structures due to mechanical impact, but no inflammatory reaction. In the lungs, fibrotic changes were observed in all infected groups, but, in the treated groups, focal lymphoplasmacytic infiltration and necrosis, characteristic of the control group, were absent. Importantly, no eosinophilic or basophilic infiltration was found in any treated group, ruling out an allergic reaction.

#### 3.2.5. Pharmacokinetics of Liposomal Phage D29

In the study on 90 mice, phage was detected exclusively in lung tissue. In samples of liver, kidneys, spleen, lymph nodes, and blood, the result was negative at all time points (Table 3). Maximum concentrations were reached at 5 min post-administration (range 1.9 × 10^3^–5.1 × 10^4^ PFU/organ). After 1 h, titres decreased by three-to-five-fold, and, by 24 h, the phage levels fell below the limit of detection (<5 × 10^3^ PFU/organ) in all animals, indicating rapid clearance. Intranasal administration provided 25–30%-higher titres compared to aerosol (*p* < 0.05). In infected animals, titres were significantly higher than in intact animals, indicating phage replication in vivo in the presence of its specific host. Multiple administrations did not lead to accumulation—titres after the course did not differ from those after a single administration. These data are consistent with the findings of Raman et al., who showed that inhaled phage administration results in effective lung accumulation with subsequent elimination over 24–72 h [30]. The absence of detectable phage in intact animals even at 5 min post-administration is attributable to two factors: (i) the rapid clearance of the liposomal formulation by the reticuloendothelial system in the absence of a permissive bacterial host, and (ii) the inability of the phage to replicate without its specific host (*M. tuberculosis*), which is absent in intact animals. In contrast, infected animals exhibited significantly higher phage titres, consistent with active in vivo replication in the presence of *M. tuberculosis*.

#### 3.2.6. Results of Toxicological Studies

The safety profile of the liposomal mycobacteriophage D29 was comprehensively evaluated in previous studies [53]. After a single 200-fold therapeutic dose (67 mg/kg lipid mass, corresponding to 5.0 × 10^8^ PFU/kg), no animal death or clinical signs of intoxication were observed. A moderate transient body weight decrease on days 2–6 was noted, which later normalised. Leukocyte levels remained within physiological range.

After a 20-day daily administration at 330 mg/kg, no lethality occurred. A statistically significant body weight decrease was observed on days 9, 11, 14–16, and 20 compared to controls, but the differences levelled off by day 28. This may reflect a non-specific reaction to the preparation.

Immunogenicity: The incubation of phage with plasma from immune animals resulted in a slight titre reduction (from 1.9 × 10^8^ to 1 × 10^8^ PFU/mL), within one order of magnitude, not impeding lytic activity. ELISA detected specific antibodies, but they did not show strong neutralising activity. These findings are consistent with previously reported results [54].

Local irritation: No redness, oedema, or papules were observed at the subcutaneous injection site, confirming the favourable local tolerability of the liposomal formulation as previously demonstrated [53].

#### 3.2.7. Histological Assessment of Local Irritation on Respiratory Mucosa

A detailed histological evaluation of the respiratory tract tissues after the aerosol and intranasal administration of the liposomal phage preparation confirmed the absence of significant local irritation. The nasal mucosa retained the normal architecture with no inflammatory changes in the aerosol group; the intranasal group showed only minimal mechanical deformation without chemical irritation. In the trachea, the treated groups lacked the lymphoplasmacytic infiltration and necrosis seen in untreated infected controls. Lung tissue showed fibrotic changes in all infected groups, but the cellular infiltration pattern differed: the controls had focal lymphoplasmacytic infiltration, whereas the treated groups lacked such infiltration, with fibrosis being comparable. Importantly, no eosinophilic or basophilic infiltrates were found, ruling out allergic reactions. Representative images are presented in Figure 2, Figure 3 and Figure 4.

## 4. Discussion

It is important to acknowledge that the individual methodological modules employed in this study (phage-based DST and liposomal phage delivery) are largely confirmatory and build upon established protocols. However, the translational value of this work resides in validating their combined application. By demonstrating that the same phage strain can reliably serve both as a diagnostic reporter and a therapeutic effector within a single workflow, this study provides a proof-of-concept for a streamlined, integrated management strategy, reducing the need for multiple distinct biological agents.

This work demonstrates the feasibility of creating a universal platform based on a single well-characterised phage strain—mycobacteriophage D29—to address two key challenges in phthisiology: the rapid diagnosis of drug resistance and the effective therapy of tuberculosis infection.

Diagnostic aspect: The AMST system combines a phenotypic approach (the assessment of *M. tuberculosis* viability in the presence of drugs) with highly sensitive molecular detection (real-time PCR). This allows the detection of resistance caused by any mechanisms, including novel and rare mutations not detected by commercial molecular tests [11,50]. The high specificity (average 96.3%) minimises false-positive results, which is critical for preserving effective drugs in the treatment regimen [55]. The time to a result—5 days—is significantly shorter than culture-based methods (up to 42–60 days) and comparable to rapid tests. The flexible configuration (three, six, or nine drugs) allows the adaptation to different clinical situations, from MDR screening to full resistance profiling. The somewhat lower sensitivity for ethambutol and bedaquiline requires further optimization (e.g., extending the drug incubation time) and confirmation on a larger set of resistant isolates. The lower sensitivity for EMB may be due to its bacteriostatic mechanism and the slow metabolic recovery of bacteria after drug exposure.

Therapeutic aspect: The liposomal phage D29 showed potent anti-mycobacterial activity in vivo. The trend towards the better efficacy of the aerosol route over the intranasal route is consistent with the literature data on the advantage of inhalation for uniform alveolar distribution [28,52,56]. Liu et al. demonstrated that the aerosol delivery of D29 provides effective airway deposition and rapid clearance, minimising systemic exposure [52]. The histological confirmation of the absence of local irritation and allergy is an important argument for the safety of the inhaled formulation.

We acknowledge that a control group receiving empty liposomes (without phage) was not included. However, previous studies from our laboratory have shown that the lipid formulation used does not exhibit antimicrobial activity in vitro [43]. Nevertheless, this represents a limitation of the present study.

Pharmacokinetics: The exclusive lung tropism (only lung accumulation) and rapid elimination (24 h) indicate a low risk of systemic side effects. These data are in line with Raman et al., who showed that inhaled phage administration results in effective lung accumulation and subsequent clearance [30]. The detection of higher phage titres in infected than in intact animals is direct evidence of phage replication in vivo in the presence of *M. tuberculosis*. This fundamental advantage—self-amplification at the infection site—allows a therapeutic effect to be achieved with lower doses and less frequent administration [44,46]. The absence of accumulation upon repeated dosing confirms the safety of the proposed regimen.

Safety: The toxicological results agree with the literature data on the lack of toxicity of bacteriophages to mammals [37,45]. The observed changes in body weight and leukocyte counts were mild and reversible, supporting further clinical development [53].

Integration of modules: The uniqueness of the work lies in creating a closed “diagnosis–therapy” cycle based on a single well-characterised phage strain. The test system allows for the rapid determination of the resistance profile, while the therapeutic preparation based on the same phage provides treatment effective even against MDR strains (confirmed in additional experiments with the clinical isolate CAO120/26 (SHRE)) [53]. This opens perspectives for a personalised approach, where, after resistance is detected, phage therapy can be initiated immediately.

It is important to note that the diagnostic results obtained with this platform are applicable exclusively to clinical isolates that support mycobacteriophage D29 replication. Our AMST methodology incorporates a built-in quality control criterion (Section 2.1.5, condition ii) that serves as a parallel screening for the phage replication capacity: if the phage fails to replicate in the growth control well (ΔCt ≤ 3), the entire test for that isolate is deemed invalid. Importantly, this screening serves a dual purpose aligned with the integrated “diagnosis-to-therapy” concept: (1) it confirms the isolate’s eligibility for phage-based diagnostic testing, and (2) it serves as a predictive biomarker for therapeutic efficacy, as the phage replication capacity in vitro is a prerequisite for effective phage therapy  in vivo. Our previous studies have demonstrated the broad lytic activity of D29 against diverse clinical *M. tuberculosis* isolates [53]. In our current study collection of 45 isolates, 100% demonstrated susceptibility to D29 and passed the built-in QC screening. However, we acknowledge that this 100% susceptibility rate is specific to our localised cohort and may not be generalizable to broader clinical populations. Future multi-centre validations utilizing larger, geographically diverse strain collections are essential for determining the true real-world host range of D29 and to accurately establish the proportion of clinical isolates eligible for this integrated diagnostic–therapeutic approach.

It is important to contextualize the scope of this proof-of-concept study within our broader research program. While the primary therapeutic efficacy evaluation in this manuscript focused on the H37Rv strain to establish baseline pharmacokinetics and acute safety, the platform’s activity against diverse clinical isolates, including MDR strains, has been explicitly demonstrated in our parallel studies [53]. Furthermore, head-to-head comparisons of this liposomal phage preparation with standard chemotherapy regimens have been successfully evaluated and presented in our recent work [57]. Finally, while the 14-day follow-up in the current study was designed to capture the acute efficacy and safety signals, further development of the platform will encompass the assessment of long-term therapeutic dynamics.

## 5. Conclusions

In this comprehensive work, an integrated platform based on mycobacteriophage D29 for the diagnosis and therapy of tuberculosis infection was successfully developed and tested.

1. The diagnostic module—the two-component AMST system with lyophilised drugs and phage DNA detection by multiplex real-time PCR—provides a phenotypic resistance profile within 5 days with a high accuracy (average sensitivity 90.0%, specificity 96.3%). The system is available in three configurations, allowing flexible adaptation to clinical needs.
2. The therapeutic module—the liposomal formulation of phage D29—demonstrates potent anti-mycobacterial activity in vivo against both the reference H37Rv strain and a clinical MDR isolate. The aerosol route showed a non-significant trend towards greater efficacy (*p* = 0.089); further studies with larger sample sizes are needed.
3. The pharmacokinetic profile is characterised by exclusive lung tropism, rapid peak concentration (5 min), complete elimination within 24 h, and no accumulation, confirming its safety.
4. The phenomenon of phage replication in vivo in the presence of its specific host was established, giving phage therapy a fundamental advantage over conventional chemotherapeutics.
5. Safety studies confirmed the absence of significant toxicity, immunogenicity, or local irritation at the selected doses and regimens.

Together with our companion studies demonstrating the efficacy against MDR strains [53] and the comparative evaluation with standard chemotherapy [57], these results provide a comprehensive preclinical foundation supporting the transition to the clinical evaluation of this integrated platform.

## Figures and Tables

**Figure 1:**
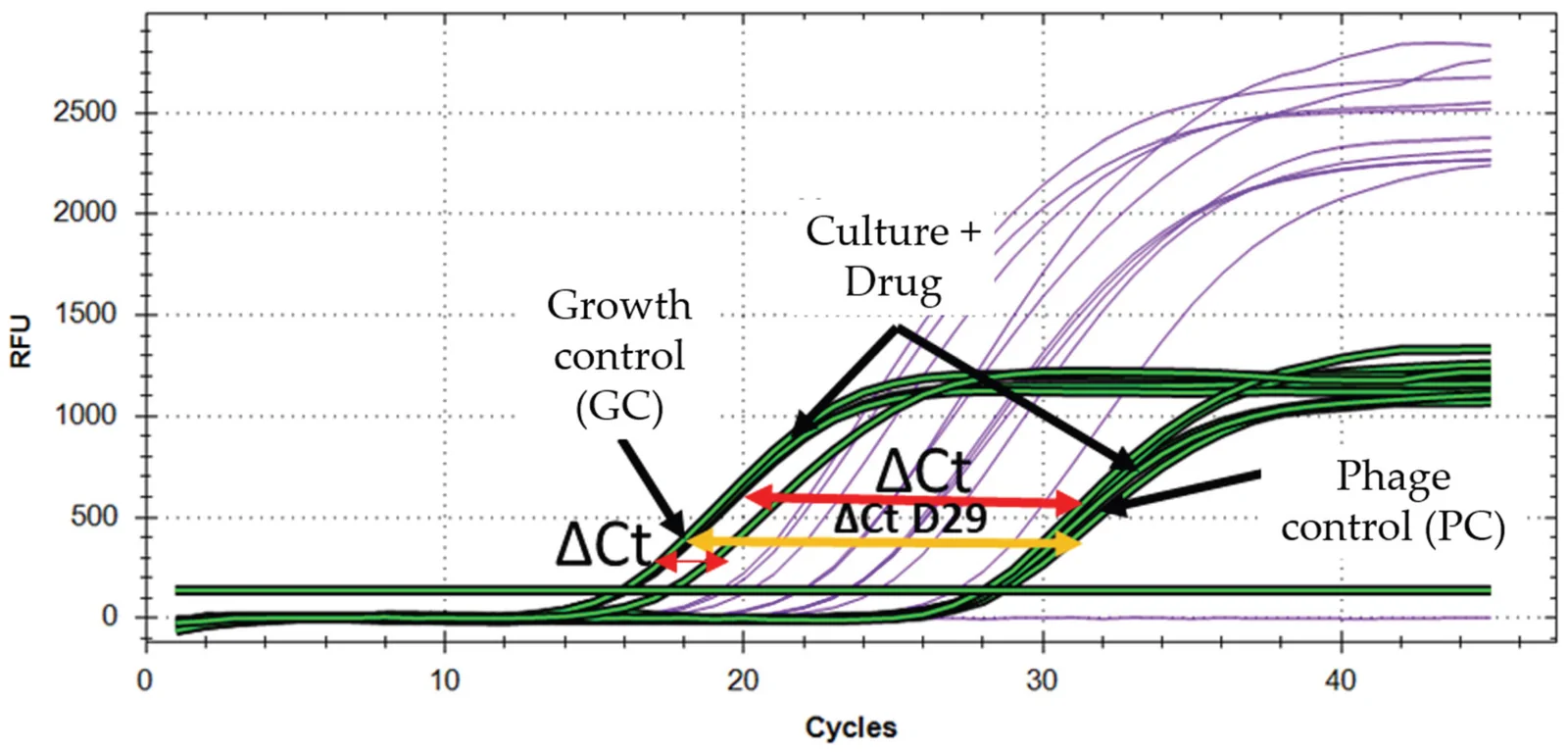
Results of analysis of *M. tuberculosis* culture No. 32: resistant to several tested drugs, retained sensitivity to one of the tested drugs. Shown are the real-time PCR amplification curves for phage D29 DNA detection (FAM channel): Culture + Drug (samples incubated with individual anti-TB drugs), Growth Control (GC, culture without drug), and Phage Control (PC, initial phage inoculum without culture). The ΔCt value is calculated as ΔCt = Ct (drug) − Ct (GC). Susceptibility is defined as ΔCt ≥ 3, resistance as ΔCt < 3.

**Figure 2:**
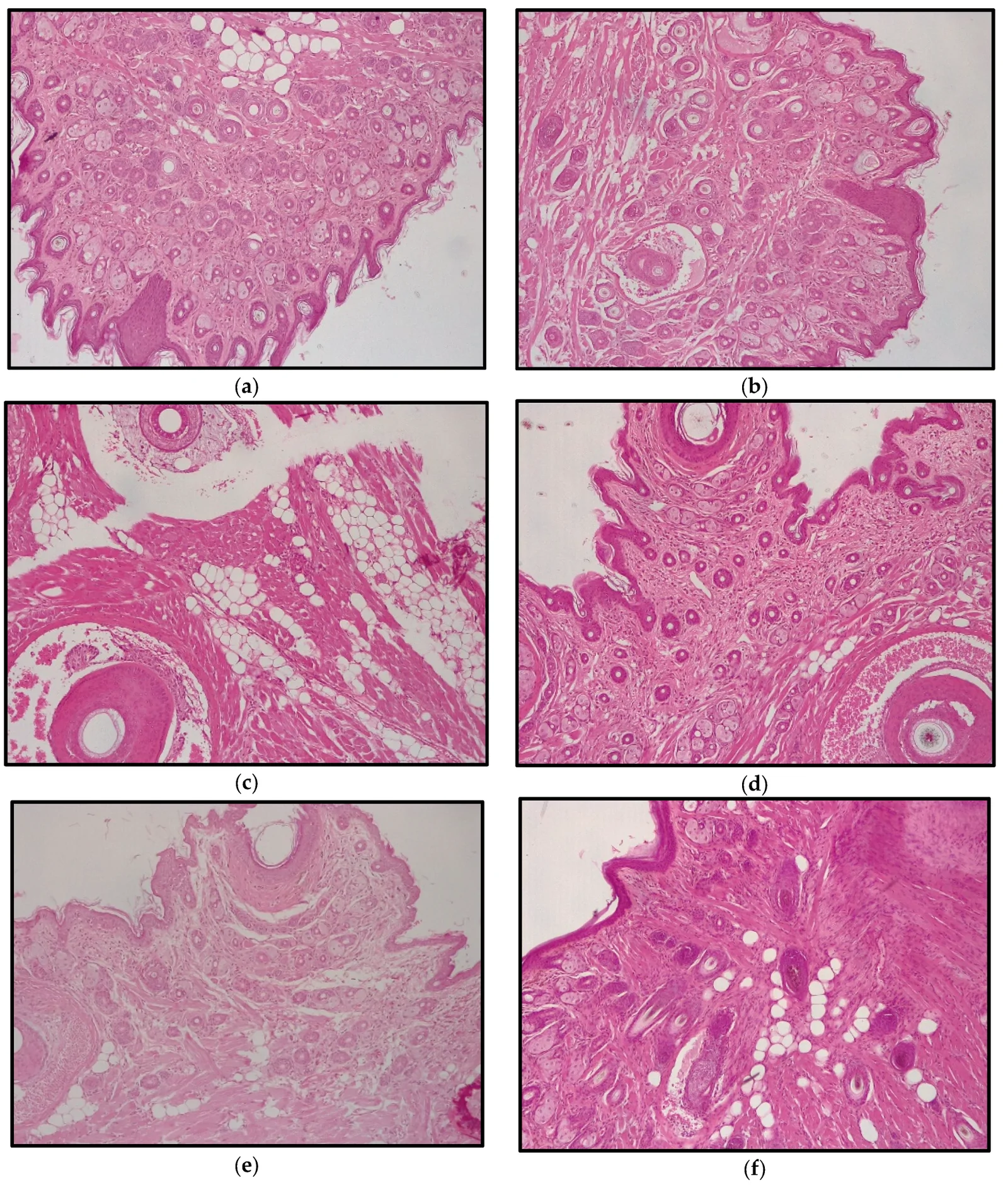
Histological examination of the nasal cavity. Aerosol administration (**a**,**b**)—normal histological structure, absence of eosinophilic and basophilic infiltration (as a marker of allergic reaction), absence of inflammatory reaction, and no haemorrhages detected. Intranasal administration (**c**,**d**)—normal histological structure, slightly deformed glands due to mechanical impact, and no haemorrhages. Control group (**e**,**f**)—infected mouse nose without drug administration—normal histological structure. Histological evaluation was performed in a blinded manner by a certified pathologist using a semi-quantitative scoring system (n = 5 animals per group).

**Figure 3:**
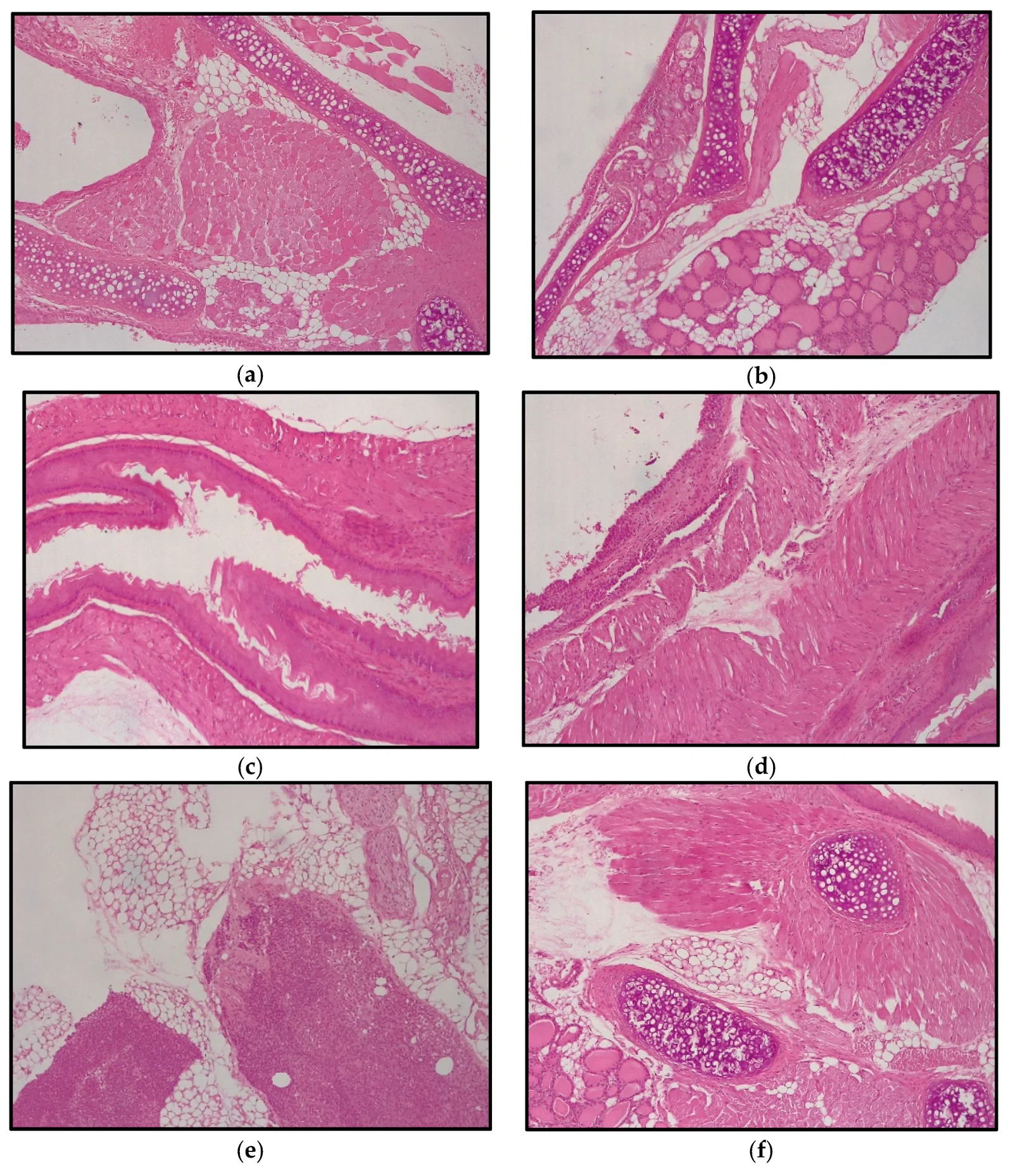
Histological examination of the trachea. Aerosol administration (**a**,**b**)—normal histological structure, absence of eosinophilic and basophilic infiltration, absence of inflammatory reaction, and no haemorrhages detected. Intranasal administration (**c**,**d**)—absence of eosinophilic and basophilic infiltration, and no haemorrhages detected. Control group (**e**,**f**)—pronounced focal lymphoplasmacytic infiltration, and areas of necrosis. Histological evaluation was performed in a blinded manner by a certified pathologist using a semi-quantitative scoring system (n = 5 animals per group).

**Figure 4:**
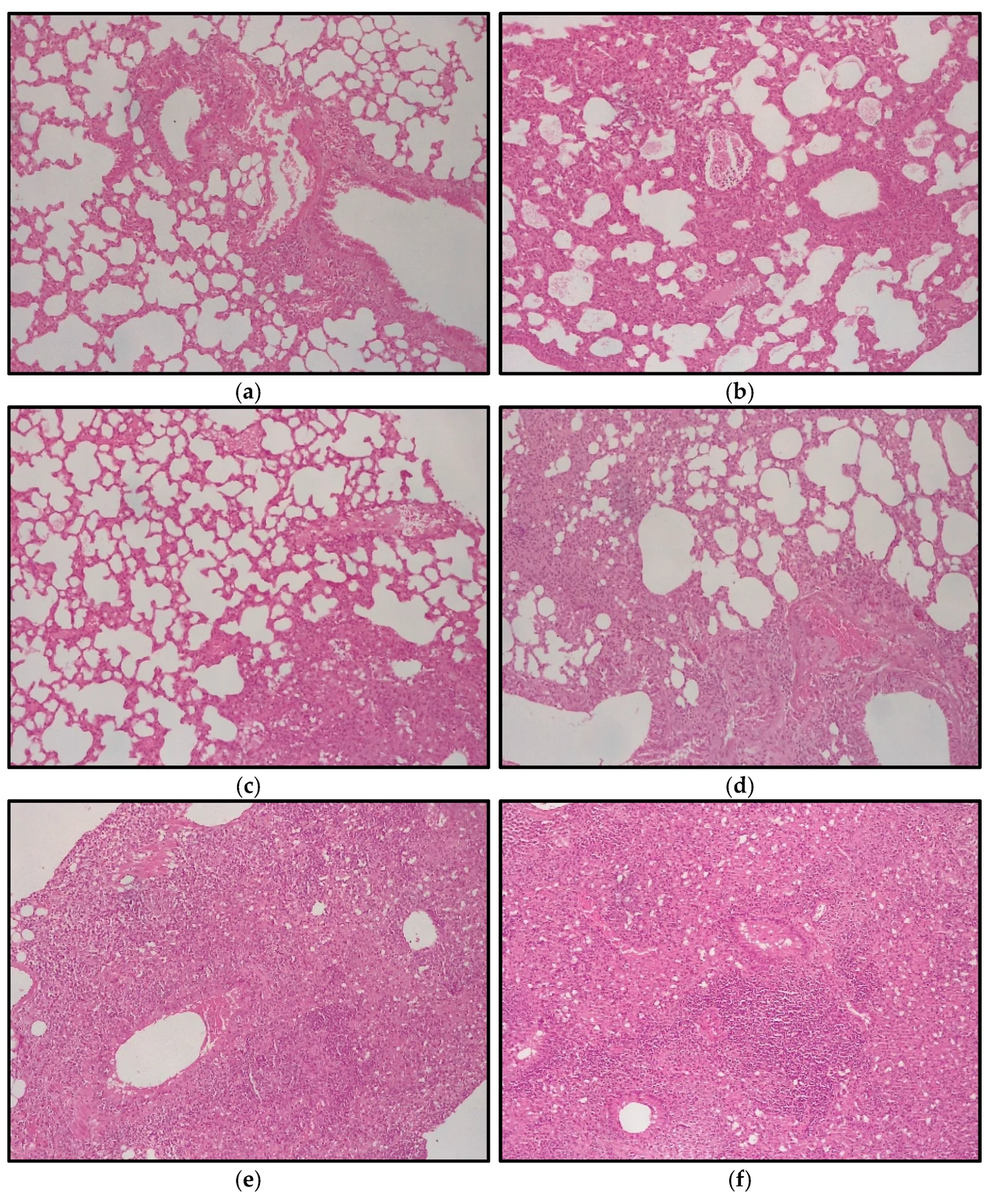
Histological examination of lung tissue. Aerosol administration (**a**,**b**)—alveolar walls slightly thickened, absence of eosinophilic and basophilic infiltration, and no haemorrhages detected. Intranasal administration (**c**,**d**)—alveolar walls slightly thickened, absence of eosinophilic and basophilic infiltration, and no haemorrhages detected. Control group (**e**,**f**)—focal lymphoplasmacytic infiltration, and areas of necrosis. Histological evaluation was performed in a blinded manner by a certified pathologist using a semi-quantitative scoring system (n = 5 animals per group).

**Table 1:** Diagnostic sensitivity and specificity of the AMST system (n = 45).

Drug	Sensitivity, % (95% CI)	Specificity, % (95% CI)
Isoniazid (INH)	97.0 (89.5–100)	94.0 (83.5–100)
Rifampicin (RIF)	96.2 (88.9–100)	89.5 (77.9–100)
Ethambutol (EMB)	71.0 (55.2–86.8)	100 (100–100)
Levofloxacin (Lfx)	95.2 (86.1–100)	100 (100–100)
Moxifloxacin (Mfx)	100 (100–100)	100 (100–100)
Bedaquiline (Bq)	67.0 (35.0–99.0)	100 (100–100)
Linezolid (Lzd)	90.0 (71.4–100)	91.4 (82.9–100)
Delamanid (Dlm)	92.0 (77.8–100)	97.0 (91.2–100)
Clofazimine (Cfz)	100 (100–100)	94.4 (87.1–100)
Average (9 drugs)	90.0	96.3

**Table 2:** Validation of the aerosol delivery system: impact of nebulisation on phage viability and liposome integrity.

Parameter	Before Nebulisation	After Nebulisation	Change
	Phage Titre (PFU/mL)		
Experiment 1	7.0 × 10^8^	2.0 × 10^8^	−0.54 log_10_
Experiment 2	5.0 × 10^8^	1.0 × 10^8^	−0.70 log_10_
Experiment 3	7.0 × 10^8^	3.0 × 10^8^	−0.37 log_10_
Mean ± SD	6.3 × 10^8^ ± 1.2 × 10^8^	2.0 × 10^8^ ± 1.0 × 10^8^	−0.54 ± 0.17 log_10_
Liposome encapsulation efficiency (%)	34.0	28.0	−6.0 percentage points

Nebuliser: OMRON NE-C300 Complete; setting 3, 0.3 mL/min, particle size 3 µm. The encapsulation efficiency was determined by qPCR using D29-specific primers and probe (Section 2.1.4).

**Table 3:** Amount of mycobacteriophage D29 (PFU/organ) in mouse lungs under different administration regimens.

Group (Condition/Route)	Time After Last Dose	Lungs (Range PFU/Organ)	Other Organs *
Intact, intranasal (multiple)	5 min	not detected	not detected
	1 h	not detected	not detected
	24 h	not detected	not detected
Intact, aerosol (multiple)	5 min	not detected	not detected
	1 h	not detected	not detected
	24 h	not detected	not detected
Infected, intranasal (multiple)	5 min	5.4 × 10^3^–3.3 × 10^4^	not detected
	1 h	2.7 × 10^3^–1.3 × 10^4^	not detected
	24 h	not detected	not detected
Infected, aerosol (multiple)	5 min	1.9 × 10^3^–3.0 × 10^4^	not detected
	1 h	4.8 × 10^3^–2.1 × 10^4^	not detected
	24 h	not detected	not detected
Infected, intranasal (single)	5 min	9.2 × 10^3^–3.8 × 10^4^	not detected
	1 h	4.0 × 10^3^–1.7 × 10^4^	not detected
	24 h	not detected	not detected
Infected, aerosol (single)	5 min	3.6 × 10^3^–5.1 × 10^4^	not detected
	1 h	3.3 × 10^3^–2.2 × 10^4^	not detected
	24 h	not detected	not detected

* Liver, kidneys, spleen, lymph nodes, blood—all negative.

## Data Availability

The original contributions presented in this study are included in the article. Further inquiries can be directed to the corresponding author.

## Abbreviations

The following abbreviations are used in this manuscript:

BAL	Bronchoalveolar lavage
Bq	Bedaquiline
CFU	Colony-forming unit
Cfz	Clofazimine
CI	Confidence interval
Dlm	Delamanid
DNA	Deoxyribonucleic acid
EMB	Ethambutol
INH	Isoniazid
Lfx	Levofloxacin
Lzd	Linezolid
MDR	Multidrug-resistant
Mfx	Moxifloxacin
OADC	Oleic acid–albumin–dextrose–catalase
PCR	Polymerase chain reaction
PFU	Plaque-forming unit
RIF	Rifampicin
TB	Tuberculosis
XDR	Extensively drug-resistant
WBCs	White blood cells
